# Attenuating Ischemia and Reperfusion Injury Using NAD^+^-Loaded Nanoparticles in Mouse Kidneys

**DOI:** 10.1097/TXD.0000000000001890

**Published:** 2025-12-12

**Authors:** Bret Verhoven, Yao Tong, Peter Chlebeck, Weixiong Zhong, Weifeng Zeng, Heather Jennings, Brooke Miller, Grace Heise, Mia Levitsky, Ruosen Xie, Shaoqin Gong, David P. Al-Adra

**Affiliations:** 1 Department of Surgery, University of Wisconsin School of Medicine and Public Health, Madison, WI.; 2 Department of Biomedical Engineering, University of Wisconsin Institute for Discovery, Madison, WI.; 3 Department of Pathology and Laboratory Medicine, University of Wisconsin School of Medicine and Public Health, Madison, WI.; 4 Pathology and Laboratory Medicine Service, William S. Middleton Memorial Veterans’ Hospital, Madison, WI.; 5 Department of Ophthalmology and Visual Sciences, University of Wisconsin, Madison, WI.; 6 Wisconsin Institute for Discovery, University of Wisconsin, Madison, WI.; 7 McPherson Eye Research Institute, University of Wisconsin, Madison, WI.

## Abstract

**Background.:**

Ischemia and reperfusion (IR) injury is a major complication in solid organ transplantation, necessitating new therapeutic strategies to suppress IR injury and thereby reduce early allograft dysfunction and delayed graft function. In kidney transplantation, IR injury is associated with disruption of mitochondrial homeostasis, including depletion of nicotinamide adenine dinucleotide (NAD^+^) leading to diminished ATP levels, which cells need for survival. Here, we describe a novel approach to attenuate IR injury in a mouse kidney model using a NAD^+^-loaded nanoparticle, capable of direct intracellular delivery of NAD^+^.

**Methods.:**

C57BL/6NCrl mice underwent a laparotomy and occlusion of the left renal pedicle for 30 min to induce IR injury. Right nephrectomy was performed during the injury incubation. Mice received the test agents by 2 different techniques: direct kidney injection via the renal artery and systemically via intravenous (IV) injection into the vena cava. Mice received a single dose of the NAD^+^-loaded nanoparticle at 3 different concentrations. Blood and kidney tissue were collected for analysis 24 h after IR injury. Measurement of blood creatinine levels and histological analysis was performed to assess the protective effectiveness of the NAD^+^-loaded nanoparticle.

**Results.:**

A single dose of NAD^+^-loaded nanoparticles resulted in significantly reduced creatinine levels in both the direct kidney-injected animals (*P* < 0.0001) and the IV-injected animals (*P* < 0.01) compared with empty nanoparticle, free NAD^+^, or saline controls. IR-induced renal tubular injury scores were markedly reduced for both IV delivery (*P* < 0.01) and direct injection (*P* < 0.05) compared with control treatments.

**Conclusions.:**

These results justify further development of this novel NAD^+^-loaded nanoparticle in the realm of organ transplantation to ameliorate IR injury and potentially increase usage of expanded criteria organs and reduce instances of delayed graft function.

## INTRODUCTION

Ischemia and reperfusion (IR) injury in kidneys, a pathological process occurring when organ blood supply is restricted and then restored, remains an intransigent challenge for kidney transplantation. Significant IR injury contributes to the development of delayed graft function (DGF) in approximately one third of all kidney transplants with this rate rising to 50% in kidneys donated after circulatory death.^1,2^ DGF is a well-established risk factor^3^ and the foremost long-term predictor for poor graft survival and necessitates increased care in the early postoperative setting waiting for the return of renal function.^4^ Therefore, there remains an unmet need for new and effective therapeutic strategies targeting IR injury in kidney transplantation.

Renal tubule cells are some of the most energy demanding cells in the body, only the heart has a higher level of mitochondria and oxygen consumption, so it is not surprising that mitochondrial dysfunction has been linked to the pathophysiology of kidney IR injury.^5,6^ Under ischemic conditions, rising calcium levels initiate the swelling of mitochondrial cristae membranes, while anaerobic metabolism depletes ATP, reduces pH, and produces reactive oxygen species (ROS). During reperfusion, reoxygenation intensifies ROS generation, triggering severe oxidative stress, which induces cell damage, unfolding of mitochondrial cristae membranes, necrotic and apoptotic cell death pathways activation, and an inflammatory immune response that culminates in acute kidney injury.^7,8^ Reduction of mitochondrial membrane potential, marked by matrix swelling and loss of cristae membranes, during IR impairs restoration of ATP production upon reperfusion.^9^ Progressive depletion of ATP during ischemia and diminished ATP synthesis upon reperfusion renders an energy collapse that hinders the cell from recovering homeostasis and initiating cell repair. In this study, we explored the possibility of targeting this cellular energy crisis as a potential avenue for IR injury therapy.

The electron carrier nicotinamide adenine dinucleotide (NAD^+^) is a critical cofactor in multiple cellular processes including the redox generation of ATP, and the nonredox NAD^+^-dependent activities of multiple enzymes including poly (ADP-ribose) polymerase (PARP), CD38, and sirtuins.^10^ Given that NAD^+^ directly or indirectly influences key cellular functions like energy metabolism, DNA repair, gene expression, and immune cell function, maintaining cellular pools of NAD^+^ is imperative for maintaining tissue homeostasis. Normally, NAD^+^ is constantly being synthesized, metabolized, and recycled to maintain relatively stable concentrations in the cytoplasm, mitochondria, and nucleus,^11^ but during IR injury, NAD^+^ supplies are significantly depleted.^12,13^ Dwindling stocks of NAD^+^ contribute directly to the cellular energy crisis experienced during IR injury and, consequently, provide a potential target for replenishment therapy.

Convincing evidence has been reported supporting the hypothesis that NAD^+^ boosting is a viable strategy for treatment of IR injury in several different organs. In preclinical studies of the kidney,^12,13^ heart,^14^ and brain,^15^ exogenous treatment with NAD^+^, its redox couple NADH, or their precursors, nicotinamide, and nicotinamide riboside, was shown to mitigate IR injury when applied pre- and/or post-injury. However, a noteworthy inadequacy of exogenous delivery of these molecules is diminished NAD^+^ bioavailability resulting from not having direct access to the cytosol.

With the aim of enhancing NAD^+^ bioavailability during repletion therapy, we engineered a NAD^+^-loaded lipid-coated nanoparticle (NAD^+^-NP) that is taken up by the cell via endocytosis, thereby replenishing cytosolic NAD^+^ through direct intracellular delivery without the requirement of precursor conversion. Previously, we have demonstrated the effectiveness of this NAD^+^-NP to ameliorate sepsis and intimal hyperplasia in murine models.^16,17^ In this study, we show that this method also attenuates IR injury in mouse kidneys following prolonged warm IR. Providing this therapy in the peritransplant environment holds the promise of increased utilization of expanded criteria organs, improved transplant outcomes, and the potential to alleviate DGF.

## MATERIALS AND METHODS

### Animals

Male C57BL/6NCrl mice (Charles River Laboratories) weighing 22–33 g (6–18 wk) were used for all experiments. Animals were housed in specific pathogen-free conditions in the animal care facilities at the University of Wisconsin-Madison Institute for Medical Research in accordance with institutional guidelines. The study protocol (No. B0007) was approved by the Institutional Animal and Use Committee at the University of Wisconsin School of Medicine and Public Health, and all animals were treated ethically.

### Materials

Calcium chloride, NAD^+^, and poly (oxyethylene)nonylphenyl ether (a nonionic surfactant) were sourced from Sigma-Aldrich (St. Louis, MO). Disodium hydrogen phosphate was purchased from Dot Scientific, Inc. (Burton, MI). L-α-phosphatidylcholine and dioleoylphosphatidic acid (DOPA) and were purchased from Avanti Polar Lipids (Alabaster, AL). N-(Methylpolyoxyethylene oxycarbonyl)-1,2-distearoyl-sn-glycero-3-phosphoethanolamine was purchased from NOF America (New York, NY). Chloroform and cyclohexane were purchased from Thermo Fisher Scientific (Fitchburg, WI).

### Preparation of the Lipid-Coated NAD^+^-Loaded NP

The NAD^+^-NP was prepared using the water-in-oil reverse microemulsion technique and thin-film hydration method previously described.^16,18^ Two reverse microemulsions, A and B, were prepared, each with a total volume of 50 mL. The organic phase of both microemulsions consisted of cyclohexane and the surfactant poly (oxyethylene)nonylphenyl ether in a volume ratio of 71:29. The aqueous phase of microemulsion A contained 1 mL of a 2.5 M calcium chloride solution and 2 mg of NAD^+^. Meanwhile, the aqueous phase of microemulsion B included 1 mL of a 25 mM disodium hydrogen phosphate solution (pH 9) with 6 mg of DOPA. The 2 microemulsions were combined and stirred for 30 min. Afterward, 100 mL of ethanol was added to demulsify and remove the oil phase. The nanoparticle was then collected by centrifugation at 10 000 g for 15 min. Following sequential washing twice with ethanol and once with 70% ethanol, the nanoparticles were resuspended in chloroform containing 5.7 mg of L-α-phosphatidylcholine, 0.57 mg of cholesterol, and 0.33 mg of N-(Methylpolyoxyethylene oxycarbonyl)-1,2-distearoyl-sn-glycero-3-phosphoethanolamine. After chloroform was removed using a rotary evaporator, the resultant lipid film was hydrated with 1 mL of 10 mM Tris-HCl buffer (pH 7.4) to form the NAD^+^-NP. To produce empty nanoparticles (ENPs), the same process was followed, without addition of NAD^+^.

### Characterization of NAD^+^-NP

The hydrodynamic diameter and zeta potential for the NAD^+^-NPs were assessed using dynamic light scattering, using a ZetaSizer Nano ZS90 spectrometer (Malvern Instruments). Morphological characteristics were examined through transmission electron microscopy on an FEI Tecnai G2 F30 TWIN 300 KV system (E.A. Fischione Instruments, Inc.).

### Surgical and Experimental Procedure

**FIGURE 1.**
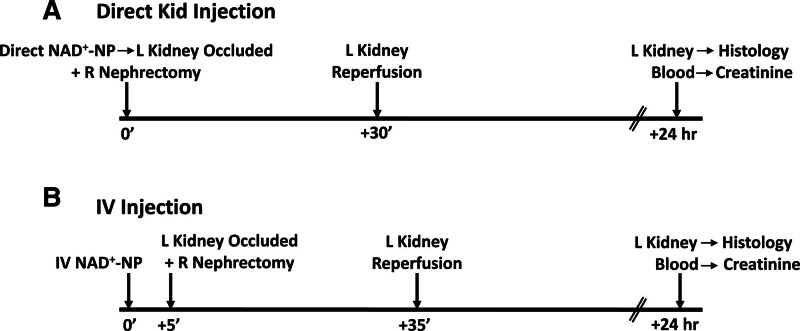
Mouse kidney ischemia-reperfusion injury (IRI) study experimental design. A, Schedule of direct kidney injection surgery. B, Schedule of IV injection surgery. IV, intravenous; NAD^+^, nicotinamide adenine dinucleotide; NP, nanoparticle.

Mice were randomized into 1 of 11 experimental and 2 control groups (Table 1), each consisting of 4 to 10 animals. NAD^+^-NP, ENP, or free NAD^+^ treatments were administered using 2 different techniques: direct injection into the kidney via the renal artery and systemically via intravenous (IV) injection of the vena cava. The schedule by which each technique was administered is shown diagrammatically in Figure 1.

**TABLE 1.** Mouse kidney NAD^+^-NP IRI study control and experimental groups

Treatment	Technique	Concentration	IRI	n
Sham	NA	NA	No	4
Saline	Kidney injection	NA	Yes	8
NAD^+^-NP	Kidney injection	2.5, 5.0, and 20 mg/kg	Yes	9, 6, 3
ENP	Kidney injection	2.5 mg/kg	Yes	4
NAD^+^	Kidney injection	2.5 and 5.0 mg/kg	Yes	7
NAD^+^-NP	IV injection	20 mg/kg	Yes	4
ENP	IV injection	20 mg/kg	Yes	5
NAD^+^	IV injection	20 mg/kg	Yes	5

NA, not applicable; ENP, empty nanoparticle; IRI, ischemia-reperfusion injury; IV, intravenous; NAD^+^, nicotinamide adenine dinucleotide; NP, nanoparticle.

Both treatment methods began with anesthetizing the mouse with 5% isoflurane inhalation, shaving the abdomen with an electric clipper and disinfecting the abdomen with 75% alcohol. The mouse was then positioned in a supine position on a heated surgery pad with its nose in an anesthesia cone and limbs immobilized with tape followed by lowering the isoflurane to 2%.

Following immobilization on the surgery pad, mice in the kidney-injected treatment group received a longitudinal midline skin and muscle incision from the pubis to the xiphoid followed by retractor insertion. The intestines were mobilized to the right side of the abdomen and covered with moistened gauze, leaving the left kidney, renal vessels, and aorta exposed. In the sham group, this was the extent of dissection and the abdominal incision was then sutured closed. For the normal saline control and experimental groups, the tips of 2 microvascular clamps were placed on the aorta as far proximal and distal to the renal vessels as possible. Normal saline, NAD^+^-NP, ENP or free NAD^+^ were raised in 100 µL of saline at concentrations of 2.5, 5.0, or 20 mg/kg and injected into the aorta distal to the renal artery. Successful injection and perfusion of the kidney was judged by the change in color of the kidney from normal red to olive green. Immediately after perfusion, a nontraumatic microvascular clamp was used to occlude the renal hilum as close to the kidney as possible, preventing blood circulation through the kidney, for a 30 min IR injury incubation. A single 11-0 suture was used to repair the needle hole in the aorta followed by removal of the micro clamps on the aorta.

Mice in the systemic IV-injected treatment group had their left kidney exposed as described above and were injected IV with 20 mg/kg NAD^+^-NP, ENP, or free NAD^+^ in 100 µL of saline via the inferior vena cava. After a 5-min incubation, to allow circulation of the NP’s, a nontraumatic microvascular clamp was used to occlude the renal hilum as close to the kidney as possible, preventing blood circulation through the kidney, for a 30-min IR injury incubation.

During their 30-min IR injury incubation, all animals received a right nephrectomy. At the end of the ischemic period, the renal hilum clamp was removed and reperfusion of the kidney confirmed visually. The abdominal incision was then sutured closed. Twenty-four hours later, the animal was anesthetized, euthanized by blood collection from a cardiac puncture, and a left nephrectomy was performed. Blood creatinine was measured on a VetScan i-STAT 1 Analyzer MN: 300 V (Abbott Park, IL) using Abbott CHEM8+ assay chips (Abbott Park, IL). The left kidney was portioned and samples preserved for histology.

Animals with surgical/procedural complications—for example, IR injury was observed incomplete, animal failed to recover from anesthesia or became moribund before experimental termination—were excluded from analysis. These events were rare (<5 across all groups) and most events occurred before the administration of the intervention; therefore, we did not include them in the analysis. The differing number of animals in experimental groups was an effort to minimize group sizes to prevent unnecessary use and euthanize of experimental animals while retaining statistical power, in line with the ethical standards required by our animal protocol.

### Histology

Kidneys were fixed in 4% paraformaldehyde and embedded in paraffin, with sections mounted onto slides and stained with hematoxylin and eosin. The renal tubular injury of each specimen was evaluated by a blinded, board-certified renal pathologist and scored on a scale of 0–5 according to the extent and severity of injury as previously described.^19^

### Statistics

Statistical analyses were performed using a 1-way ANOVA with subsequent Tukey’s multiple comparisons test using GraphPad Prism v.10.0.03 software. Data are expressed as mean and confidence intervals.

## RESULTS

### Synthesis and Characterization of NAD^+^-NP

**FIGURE 2.**
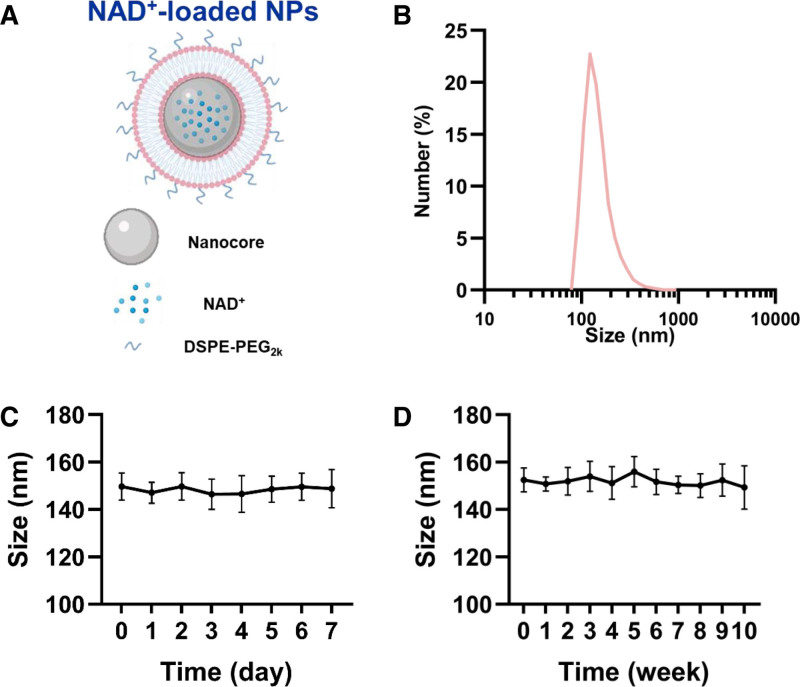
Nanoparticle characterization. A, Schematic illustration of the nanoparticle structure. B, Hydrodynamic size of NAD^+^-NP measured by DLS. C, Hydrodynamic size of NAD^+^-NP dispersed in aqueous solution at 4 °C monitored by DLS. D, Hydrodynamic size of lyophilized NAD^+^-NP lyophilized containing 10 % sucrose as cryoprotectant and stored at –20 °C. DLS, dynamic light scattering; DSPE-PEG_2k_, N-(Methylpolyoxyethylene oxycarbonyl)-1,2-distearoyl-sn-glycero-3-phosphoethanolamine; NAD^+^, nicotinamide adenine dinucleotide; NP, nanoparticle.

A schematic illustration of the nanoparticle structure is shown in Figure 2A. Nanoparticles had an average hydrodynamic diameter of approximately 150 nm (Figure 2B) and zeta potential of about –6.5 mV. Stability studies indicated that NAD^+^-NP are stable at 4 °C for 1 wk (Figure 2C). In addition, lyophilized NAD^+^-NP retained stability for up to 10 wk stored at –20 °C, as demonstrated in Figure 2D.

### NAD^+^-NP Improved Renal Function

**FIGURE 3.**
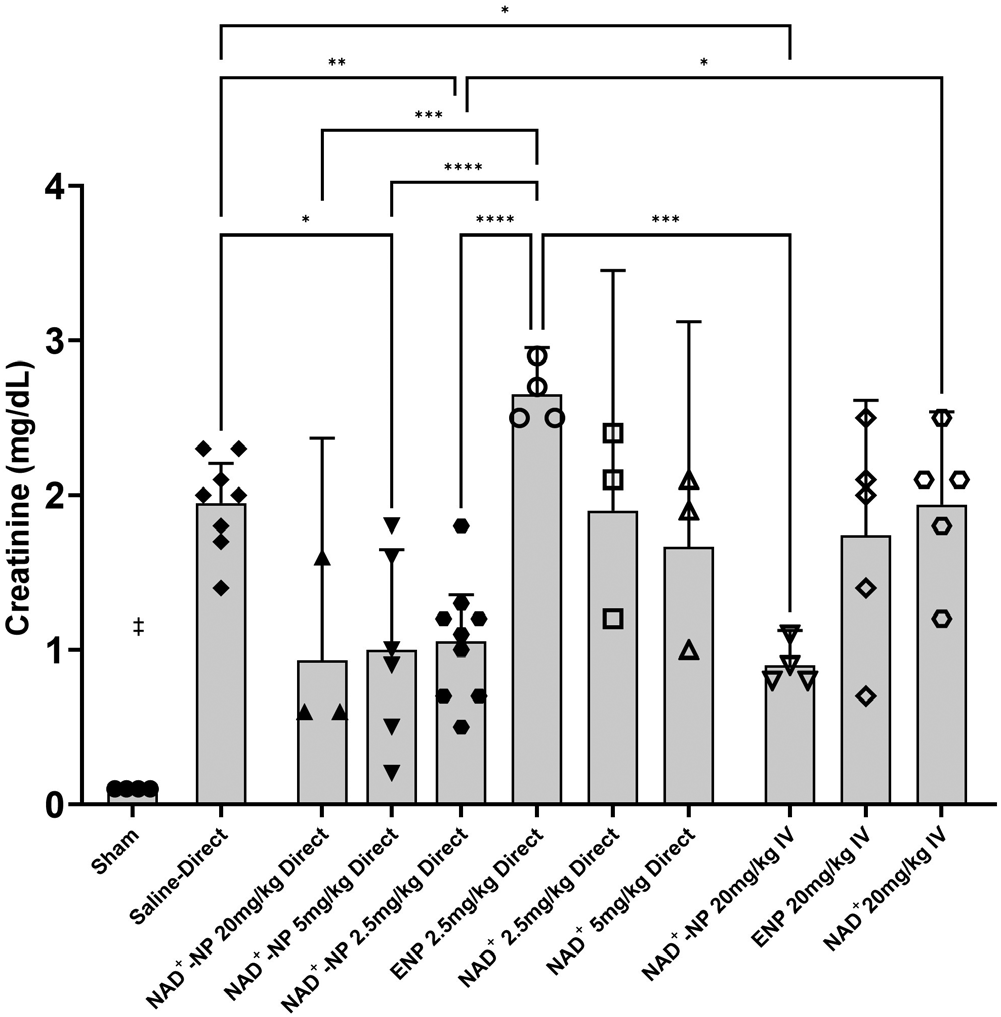
Blood creatinine in experimental groups. Graph comparing blood creatinine values for all treatment groups. Statistical analysis performed via Tukey’s comparisons test. *P < 0.05, **P< 0.01, ***P < 0.001, ****P < 0.0001. ‡Sham values were significantly different in multiple comparison tests compared with saline-direct, ENP 2.5 mg/kg direct, NAD^+^ direct, NAD^+^ 20 mg/kg IV, *P* < 0.0001; ENP 20 mg/kg IV, NAD^+^ 2.5 mg/kg direct, *P* < 0.001; NAD^+^ 2.5 mg/kg direct, *P* < 0.01; NAD^+^-NP 2.5 mg/kg direct, *P* < 0.05; and Sham values were not significantly different (*P* > 0.05) compared with remaining groups. ENP, empty nanoparticle; IV, intravenous; NAD^+^, nicotinamide adenine dinucleotide; NP, nanoparticle.

The ability of NAD^+^-loaded nanoparticles to protect against IR injury was tested using 2 different injection techniques, direct kidney injection or IV, before clamping the left kidney hilum. Both methods were effective at reducing blood creatinine levels 24 h after IR injury. Animals that received NAD^+^-NP via direct kidney injection, in all 3 concentrations, demonstrated a significant decrease in blood creatinine after 24 h compared with animals injected with ENP (Figure 3; 2.5 and 5.0 mg/kg, *P* < 0.0001; 20 mg/kg, *P* < 0.001). In addition, animals treated with direct injection of 2.5 or 5.0 mg/kg NAD^+^-NP had significantly lower blood creatinine compared with control animals injected with normal saline (Figure 3; 2.5 mg/kg, *P* < 0.01; 5.0 mg/kg, *P* < 0.05). Likewise, mice that received 20 mg/kg NAD^+^-NP IV also showed significantly reduced levels of creatinine compared with control mice injected IV with only free NAD^+^ (Figure 3; *P* < 0.05).

### NAD^+^-NP Reduced Renal Damage

**FIGURE 4.**
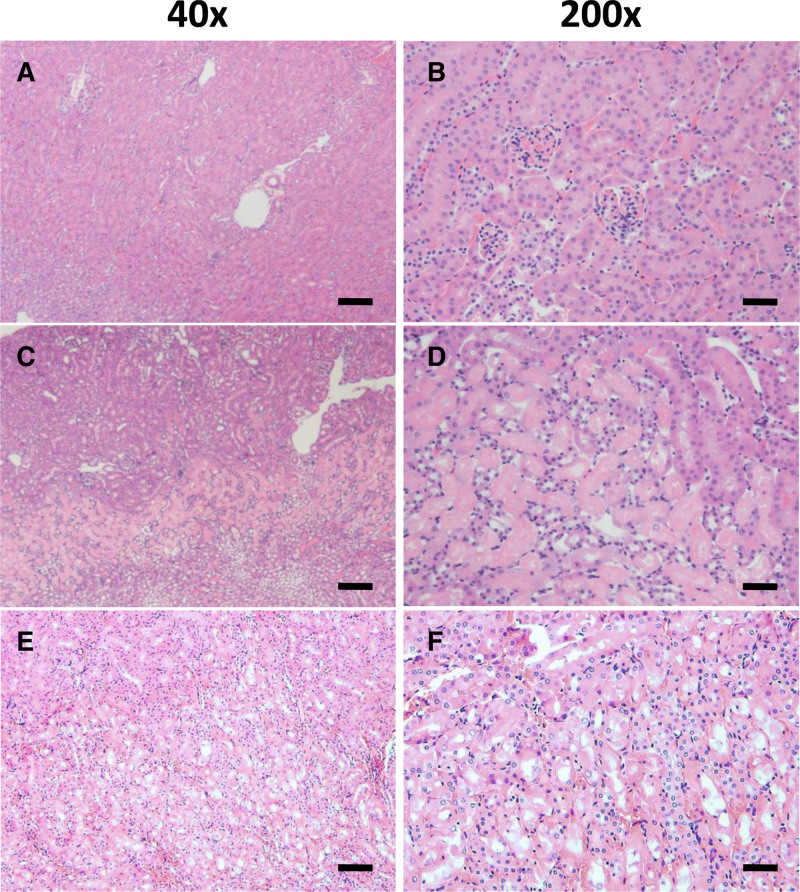
Kidney histology. Representative kidney H&E micrographs of sham (A and B), empty nanoparticle (C and D), and NAD^+^-loaded nanoparticle (E and F) treated mice. Scale bars: 250 μm (A, C, E) and 50 μm (B, D, F). H&E, hematoxylin and eosin; NAD^+^, nicotinamide adenine dinucleotide.

Kidneys were retrieved for histological analysis 24 h after IR injury. Kidney histology of the sham surgery animals was normal (Figure 4A and B). However, substantial renal changes in control mice that received saline, ENP, or free NAD^+^ were observed, including loss of tubule brush borders, tubule luminal congestion, and tubule denuclearization (Figure 4C and D). In contrast, renal histology of NAD^+^-NP’s treated mice demonstrated near normal brush borders in slightly dilated tubules but no luminal congestion or denuclearization (Figure 4E and F; representative histology pictures from the remaining groups not pictured in Figure 4 are included as **Figure S1** [**SDC**, https://links.lww.com/TXD/A813]).

**FIGURE 5.**
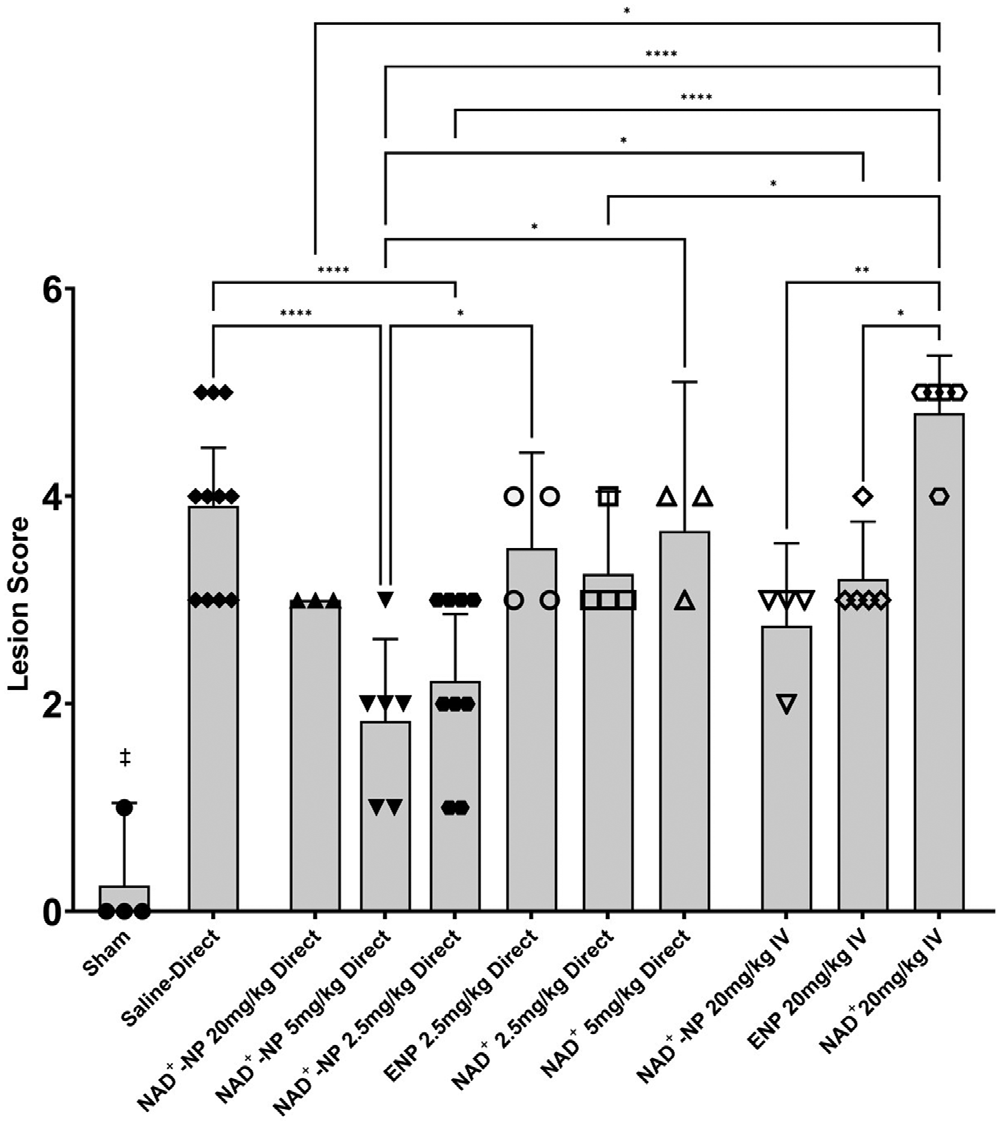
Histological lesion score. Graph comparing tubule lesion scores for all treatment groups. Statistical analysis performed via Tukey’s comparisons test, *P < 0.05, **P < 0.01, ****P < 0.0001. ‡Sham values were significantly different from all groups in multiple comparison tests (*P* < 0.05 vs NAD^+^-NP 5 mg/kg direct; *P* < 0.001 vs NAD^+^-NP 2.5 mg/kg direct; NAD^+^-NP 20 mg/kg direct, NAD^+^-NP 20 mg/kg IV; *P* < 0.0001 vs all other groups). ENP, empty nanoparticle; IV, intravenous; NAD^+^, nicotinamide adenine dinucleotide; NP, nanoparticle.

Pathological evaluation of mice that received NAD^+^-NP via direct kidney injection at concentrations of 2.5 and 5.0 mg/kg demonstrated a significant decrease in lesion score compared with animals injected with ENP (Figure 5). Likewise, mice that received 20 mg/kg NAD^+^-NP IV also showed significantly reduced lesion scores compared with control mice injected IV with only free NAD^+^ (Figure 5).

## DISCUSSION

IR injury is a universal problem for all solid organ transplants, including kidneys where it is a strong risk factor for DGF that is seen in up to 50% of transplanted kidneys.^1,2^ We undertook this study to determine if NAD^+^-loaded nanocarriers, that enable direct intracellular delivery of NAD^+^ in vivo, would be effective in attenuating the IR-induced damage that follows prolonged warm IR in a murine kidney model. Our data demonstrates that direct delivery to the kidney of NAD^+^-loaded NPs at dosages as low as 2.5 mg/kg to kidneys before warm ischemia followed by reperfusion can ameliorate injury and promote recovery to near normal functionality when measured by serum creatinine levels and histopathological evaluation of lesion scores. Furthermore, we found similar protective outcomes using the NAD^+^-loaded nanocarrier at 20 mg/kg delivered systemically by IV injection. In both systemic and direct treatment modalities, empty NP’s and free NAD^+^ displayed no effect at mitigating IR injury. The doses chosen in the current article were informed by our prior results in a sepsis model where we used NAD+ NP at 20 mg/kg administered systemically.^16^ Since 20 mg/kg was used systemically, we started the dose titrations at almost 1/10th (2.5 mg/kg) this amount for the direct injection group. In addition, the doses used in the current experiments were informed by a smaller dose-response experiment; IV treatment with free NAD+ at a high concentration (20 mg/kg) and direct injection of free NAD+ at 5 mg/kg both resulted in worse kidney injury as well as creatinine scores than the saline control, demonstrating a toxic effect (data not shown).

Several studies, in different animal models, have demonstrated that exogenous NAD^+^ boosting inhibits IR injury.^12-15^ These studies are valuable proof of concept for this strategy; however, bioavailability after exogenous delivery is constrained by NAD^+^ and its precursors not having direct access to the cytosol. Passive transmembrane diffusion of NAD^+^ has been found only in CD38^+^ cells with elevated extracellular NAD^+^ concentrations.^20^ At physiological concentrations, NAD^+^, NADH, nicotinamide, and nicotinamide riboside require active transport to enter the cell.^21^ Furthermore, reports demonstrate that NAD^+^ is routinely degraded extracellularly into its precursors before being carried into the cell^22^ and, once inside the cell, precursors still need to be converted back to NAD^+^ before they can afford full bioavailability. The limitations of exogenous delivery would require NAD^+^ repletion therapies to use dosages and treatment frequencies beyond the level of clinical usefulness in humans.^23^ In addition, we saw detrimental kidney function and lesion score when using high-dose free NAD+ delivered either via direct injection into the kidney or systemically via IV (data not shown). The innovative NAD^+^-loaded NP used in this study overcomes these limitations by enabling direct intracellular delivery through the endocytic pathway while protecting NAD^+^ from degradation outside the cell. In addition, nanoparticles offer the potential for targeted delivery by having kidney-specific targeting peptides added to their surface^24,25^ or by cloaking them in a platelet biomimetic surface, producing adherence in a variety of disease environments.^16,26^ Disease and organ-targeting NP’s provide a potential new approach for therapeutics delivery using significantly reduced effective drug doses. These methods would also allow renal targeted NAD^+^-NP’s to be given IV to the donor (before kidney donation) and to the recipient (postsurgery) providing an uninterrupted continuation of NAD^+^ augmentation therapy.

The depletion of intracellular NAD^+^ observed during IR injury occurs as a consequence of accelerated consumption and diminished biosynthesis.^27^ There are 3 pivotal NAD^+^-dependent enzymes competing for NAD^+^ in IR damaged cells.^27,28^ The first are the sirtuins, which are protein activation regulators that are NAD^+^-dependent histone deacetylases that use 30% of cellular NAD^+^ for their basal activity under normal homeostatic conditions. Moreover, recovering homeostasis after an ischemic insult requires even higher levels of sirtuin activity. The second major consumption of NAD^+^ related to IR injury results from a pathological activation of PARP1. PARP1 is a DNA damage recognition enzyme that uses large amounts of NAD^+^ and when over activated, consumes NAD^+^ at levels capable of inhibiting the renal protective benefits of sirtuin activity. The third major consumer of NAD^+^ activated during IR is CD38. Recognized as the cells primary NAD glycohydrolase, CD38 uses large amounts of NAD^+^ as substrate to generate second messengers for calcium signaling promoting cell death. Diminished biosynthesis of NAD^+^ occurs in the mitochondria, where falling NAD^+^ levels during IR impede fatty acid oxidation resulting in reduced ATP generation.^12^ Studies have shown that IR suppresses the mitochondrial biogenesis regulator peroxisome proliferator-activated receptor gamma coactivator 1-alpha, which coordinates the de novo NAD^+^ biosynthesis pathway, resulting in the reduction of NAD^+^ synthesis.^12,29^ This complex interconnected network of competing NAD^+^-dependent redox and enzymatic reactions elucidates how diversity of function amplifies the cellular collapse of NAD^+^ and shifts metabolic signaling during IR injury, making the availability of NAD^+^ itself a key regulator of NAD^+^-dependent enzymes and ultimately cell homeostasis.

Our study has several limitations. First, this was a pilot study without performing kidney transplantation. Therefore, the methods of NAD^+^-NP delivery and the effects after transplantation may be different when transplant surgery is performed. Second, although we used an inbred mice strain, genetic and age diversity regarding molecular response mechanisms to IR injury may be present, especially given the complex and interconnected molecular pathways involved. Last, small animal experiments may not translate into larger animal or preclinical models.

Future studies will investigate the mechanisms by which NAD+ leads to renal protection in this IR injury model. Specifically, we are looking into the effects on the electron transport chain using high-resolution respirometry in mitochondria isolated from the kidneys. In addition, we are going to determine the therapeutic effects of NAD^+^-NP therapy on donor kidneys in a syngeneic kidney transplant model. This will allow the assessment of the ability of NAD^+^-NP administration to decrease IR injury in an in vivo transplant model. The addition of NAD^+^-NP to organ preservation solution (either static cold storage or machine perfusion) may also offer an important therapeutic window. In addition, our laboratory has experience in organ transplantation using a novel ROS scavenger, PrC-210, and it would be advantageous to determine if the effects of increased NAD^+^ delivery combined with ROS scavenging are complementary.^30^

In conclusion, we demonstrate the ability of NAD^+^-NP’s to decrease IR injury in a kidney model, which may improve outcomes and reduce DGF when applied in a transplant model. This intimates that NAD^+^-NP’s might confer broadly applicable organ protection from IR injury in a variety of transplant and surgical settings.

## Supplementary Material


