# Supplementary figures and images for: Attenuating Ischemia and Reperfusion Injury Using NAD+-Loaded Nanoparticles in Mouse Kidneys
Source: Transplant Direct. 2025 Dec 12;12(1):e1890. doi: 10.1097/TXD.0000000000001890 (PMC13340656; doi:10.1097/TXD.0000000000001890)

**40x**

**200x**

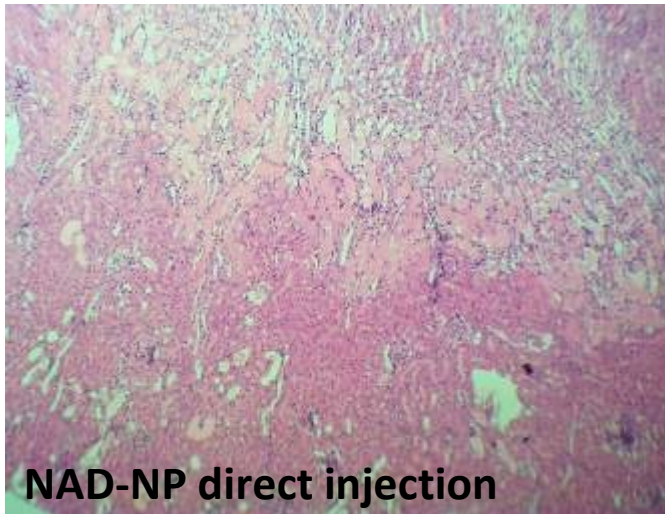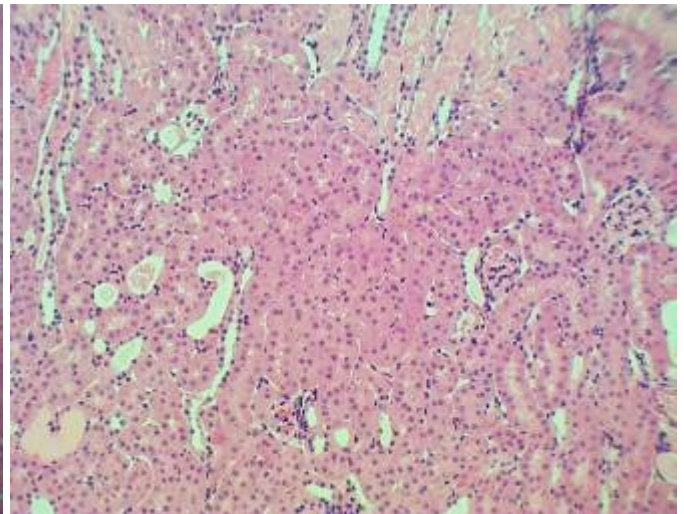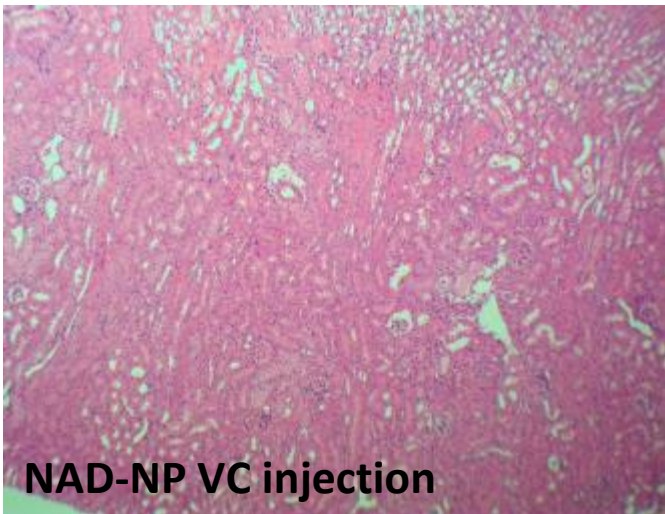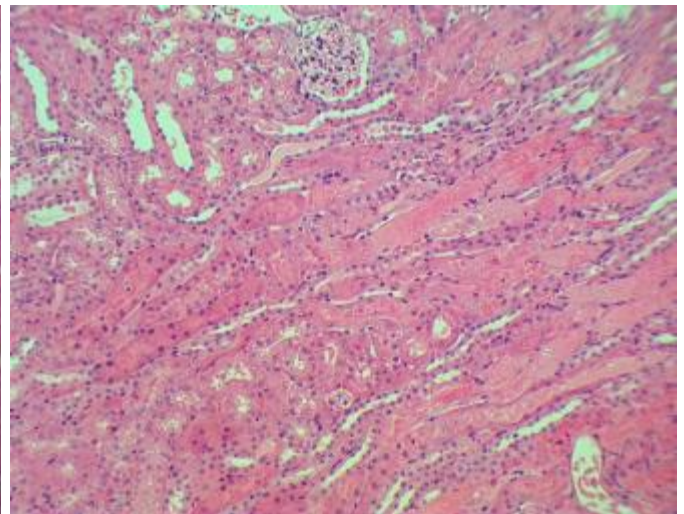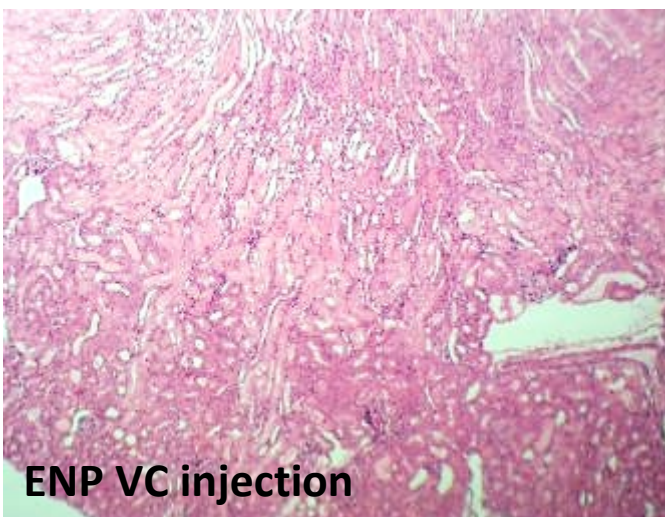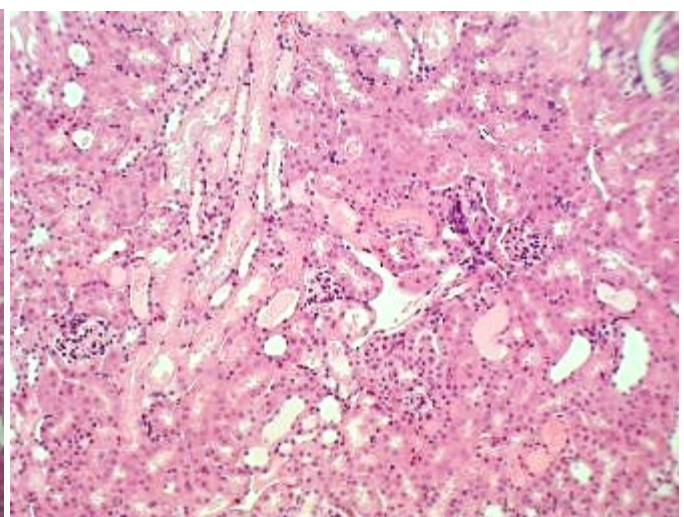

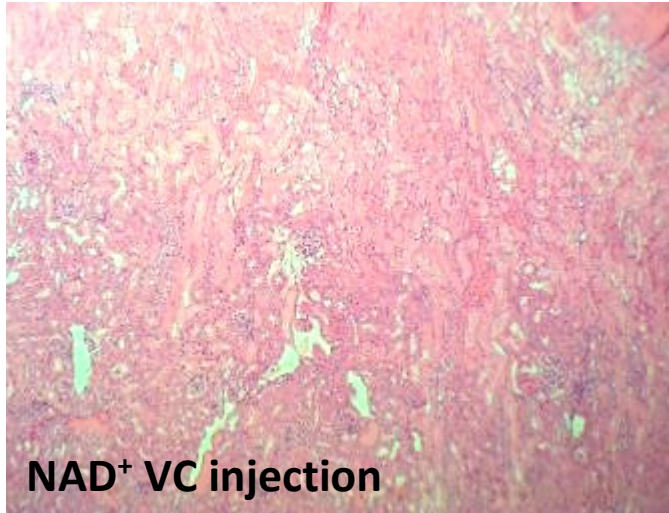

**NAD<sup>+</sup> VC injection**

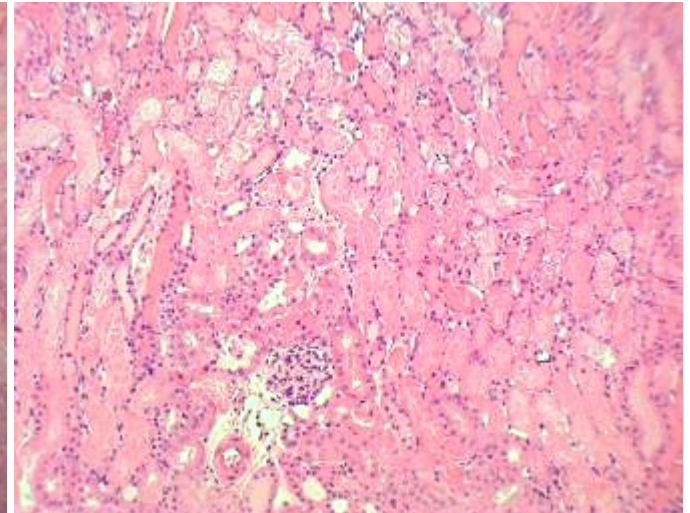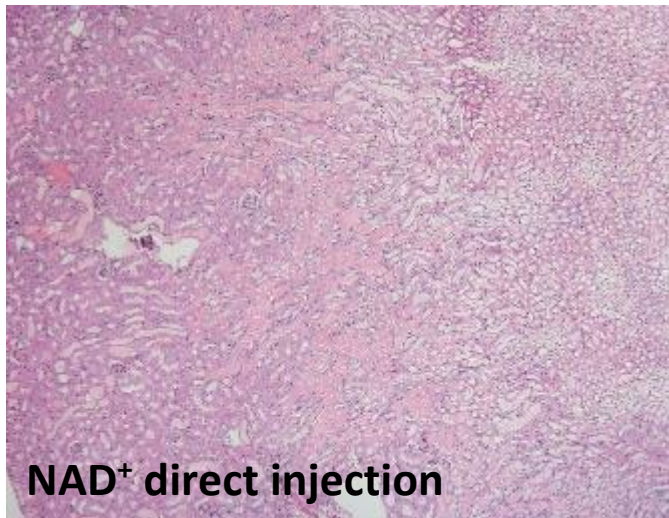

**NAD<sup>+</sup> direct injection**

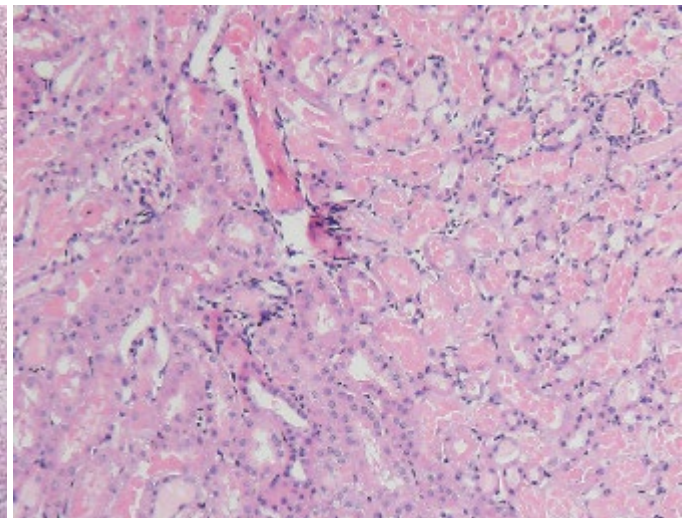

Supplement: Supplementary file 1 [file txd-12-e1890-s001.pdf]
